# Porous α-Fe_2_O_3_ nanocarriers: Biosynthesis and *in vitro* gene delivery applications

**DOI:** 10.1016/j.heliyon.2024.e28676

**Published:** 2024-04-02

**Authors:** Hajar Q. Alijani, Shahram Pourseyedi, Masoud Torkzadeh-Mahani, Mehrdad Khatami

**Affiliations:** a Research and Technology Institute of Plant Production, Shahid Bahonar University of Kerman, Kerman, Iran; b Department of Biotechnology, Shahid Bahonar University of Kerman, Kerman, Iran; c Department of Biotechnology, Institute of Science and High Technology and Environmental Sciences, Graduate University of Advanced Technology, Kerman, Iran; d Department of Medical Biotechnology, Faculty of Medical Sciences, Tarbiat Modares University, Tehran, Iran

**Keywords:** Non-viral transfection, *In vitro* toxicity, Porous α-Fe_2_O_3_ nanoparticle, Green nanotechnology

## Abstract

Non-viral gene delivery is a new therapeutic in the treating genetic disorders. The most important challenge in nonviral gene transformation is the immunogenicity of carriers. Nowadays, The immunogenicity of nanocarriers as a deliverer of nucleic acid molecules has received significant attention. In this research, hematite green nanocarriers were prepared in one step with rosemary extract. Synthetic nanocarriers were investigated by using XRD (X-ray diffraction analysis), FESEM-EDX (field emission scanning electron microscopy with energy dispersive X-Ray spectroscopy), HR-TEM (high-resolution transmission electron microscopy), VSM (value stream mapping), TGA- DTG (thermal gravimetric analysis-differential thermal analysis), FT-IR (fourier-transform infrared spectroscopy), BET (brunauer–emmett–teller) and BJH (barrett-joyner-halenda) analyses. The cytotoxicity of synthetic nanocarriers was evaluated on HEK-293Tcell lines at concentration of 1–500 μg/ml using MTT method. Finally, targeted transfection of GFP plasmid using green porous particles was performed using an external magnetic field. Biogenic hematite nanoparticles with hexagonal crystal structures have a 3D pile flower-like morphology. The existence of rosemary phytochemicals in the construction of nanoparticles has caused minimal toxicity and high biocompatibility of nanocarriers. Also, TGA studies confirmed the stability of bionic nanoparticles. Superparamagnetic green nanocarriers at concentrations above 500 μg/ml is not toxic to HEK293T cells. The delivery efficiency of the plasmid was optimal at an N/P ratio of 3. Therefore, the porous α-Fe_2_O_3_ green nanocarriers are non-viral and safe carriers with potential applications in gene therapy.

## 1 Introduction

The biomedical applications development of nanomaterials depends on their cellular and environmental biocompatibility [1]. Nanoparticles synthesized using green nanotechnology are well-met needs of biomedical [2,3]. Green nanotechnology uses green chemistry to produce green nanoparticles without toxic constituents at low temperatures using less energy [[4], [5], [6]]. Nanomaterials produced using green nanotechnology are stable and directly or indirectly have minimal human and environmental hazards [[7], [8], [9]]. Hence, today green nanoparticles have received much attention for their applications in the field of gene/drug therapy [10], biosensors [11,12], cancer therapy, and enzyme stability. Meanwhile, gene/drug targeted delivery reduces the dose of the drug/DNA and prevents damage to healthy cells [13]. Also, the porosity of nanoparticles leads to the elimination of the nanoparticle functionalization step using polymers in gene/drug delivery [14,15]. The surface adsorption process encapsulates the gene/drug in the pores of nanoparticles [16,17]. As a result, the cost of producing delivery systems is reduced. On the other hand, significant mortality of cancerous tumors is due to cause synergistic combined effects of mesoporous nanocarriers and drugs [[18], [19], [20], [21]].

The hydrothermal method is one of the most widely used bottom-up methods for the production of nanostructures, which has received a lot of attention today due to its simplicity and cost-effectiveness [22]. In this study, rosemary extract was used to control the morphology and nature of nanoparticles in the hydrothermal process. According to the studies, rosemary polyphenols, due to their intrinsic characteristics and eco-friendly hydrothermal synthesis method, cause the creation of various cubic, rod, polyhedral, whisker, flower, shapes and porous nature. According to the literature, in hydrothermal synthesis, the balance between the polygonal crystals of nanoparticles and their uniform size is achieved under optimal solvent conditions, at the appropriate temperature and pressure of the autoclave. Also, using a high concentration of rosemary extract causes agglomeration and the formation of large particles. Also, high calcination temperature increases the size of nanoparticles [23,24].

Iron magnetic nanoparticles are the most well-known tool in this field Three polymorphic forms of iron are known α, ϒ, and δ [25,26]. α-Fe_2_O_3_ is the most stable Fe oxide with the chemical formula Fe_2_O_3_ in brown, red-gray, and black. Rhombohedral-hexagonal hematite with special optical and magnetic properties [27] is used in immunomagnetic separation, sensors [28], cancer/gene therapy [29], removal of ionic dyes [30], magnetic resonance imaging (MRI), cellular labeling, and so on [31,32]. Liang et al. showed transfection efficiency of miRNA using Fe_2_O_3_ nanoparticles was significantly increased. They showed that nanocarriers PEI-capped Fe_2_O_3_ are not cytotoxic and are a desirable tool in gene therapy [33]. Xia et al. showed that fluorescent-magnetic α- Fe_2_O_3_ @ Y_2_O_3_: Eu^3+^ NPs have an optimal efficiency of ibuprofen loading [34]. Also α- Fe_2_O_3_ nanoparticles enhanced the anti-cancer effect of Chitosan/polyvinylpyrrolidone/α- Fe_2_O_3_ nanocomposites against MCF-7 cells [35]. Therefore, in this study, hematite α-Fe_2_O_3_ NPs green porous nanoparticles under the optimal solvent condition of DI: extract synthesized using the hydrothermal method. Green nanocarriers are characterized using XRD, SEM, HRTEM, BET, FTIR, VSM and TG- DT analyses. Then loaded GFP on porous nanocarriers, and cytotoxicity effects of GFP -porous complex were determined on HEK-293T cell lines.

## 2 Materials and method

### 2.1 Biosynthesis of porous α-Fe_2_O_3_ NPs

*Rosmarinus officinalis* fresh leaves were sterilized and dried. The aqueous extract of the leaves was made by heating at 80 °C (1 g/5 cc) and filtering with the Buchner funnel. Deionized water (DI) was used in all stages. Ferric nitrate nonahydrate (Fe (NO₃) ₃ * 9 H₂O ≥ 99.95%, Sigma-Aldrich) was dissolved in rosemary extract with a ratio of 1: 6 at 10^0^C under vigorous stirring. The resulting solution was diluted with DI to 50 cc. The resulting solution was heated in a teflon-lined autoclave at 170 °C for 14 h. The resulting nanocarrier was washed with DI. Eventually, green nanocarriers were dried at 80 °C for 16 h and calcined for 2 h at 350 °C [36].

### 2.2 Characterization of porous α-Fe_2_O_3_ NPs

The structural properties of synthetic nanopowders were determined by XRD, SEM, and HRTEM analysis [37,38]. The crystal structure of the nanopowder was evaluated using X'PertPro, a Panalytical company in the 2θ of 10°–80° with Cu- Kα (λ = 1.54 Å). Chemical compounds and nano-powder morphology was performed using FESEM- EDAX analytics (Sigma VP, ZEISS Co.). Hematite nanopowder microstructure features were performed with HRTEM (Tecnai 20, FEI Co.). Measuring magnetic properties and determining the amount of hollow space within the synthetic hematite was determined using VSM (LBKFB, Kashan Kavir Meghnatis Co.), BET (Belsorp mini II, Microtrac Bel Corp Co.), respectively. The nano powder was degassed at 200 °C prior to the BET technique, and evaluations were done with the N_2_ adsorption. TGA-DTG (TGA2, Mettler Toledo Co.) technique was used to measure changes in chemical/physical behavior of synthesized nanopowder under temperature of 25–800 °C. These measurements done with rate of 10 °C/min^−1^ in air atmospheres. FTIR technique (tensor II device, Bruker Co.) was performed to identify the bonds in extract and synthesized nanopowder in wavelength of 400–4000 cm^−1^, with KBr plate at room temperature [39].

### 2.3 MTT assay

The growth inhibition effect of nanocarriers was evaluated by MTT (C_18_H_16_BrN_5_S, Sigma-Aldrich Co.) colorimetric dye reduction method on HEK 293T (Pasteur Institute of Iran) cells. 10^4^ of HEK 293T cells were cultivated in a 96 well microplate and retained for 24 h. Various concentrations of nanocarriers (1, 5, 10, 50, 100, 250, 500 μg/ml) were transferred to microplate and were incubated [37 °C for 24 h]. In the following, 15 μL of MTT solution (4 mg/mL) was added to microplate. And then incubation in the same condition, the medium was removed, and 100 μL of DMSO (C_2_H_6_OS, dimethyl sulfoxide, Sigma-Aldrich chemical Co.) (0.5 mM) was gradually added to microplate. Finally, the absorbance was read at 490 nm by ELISA Reader (BioTek, US) [40]. Experiments were performed in triplicate and repeated three times.

### 2.4 Gene transformation

24 h before gene delivery, 2E5 cells/well of HEK 293T cells were cultivated in a 24 well microplate and were multiplied 24 h to 80%. For gene delivery, the culture media were shifted with pre-warmed PBS (phosphate buffer saline, shellmax Company. Iran) buffer for washing. The nanocarrier/DNA complex (pDNA 0.5 μg/well, N/P ratio = 3) were added to the microplate and then incubated for 6 h. A 0.3 T magnet was located under microplate for 60 min. Then the cells were cultured in DMEM (hoher Glucoseanteil, Merck) medium containing 10% FBS (Fetal bovine serum, Biochrome, Germany) and incubated in the same conditions for 24 h without magnet. Finally, the treated cells were evaluated by fluorescent microscopy [40].

## 3 Results and discussion

### 3.1 Characterization of porous α-Fe_2_O_3_ NPs

**Fig. 1:**
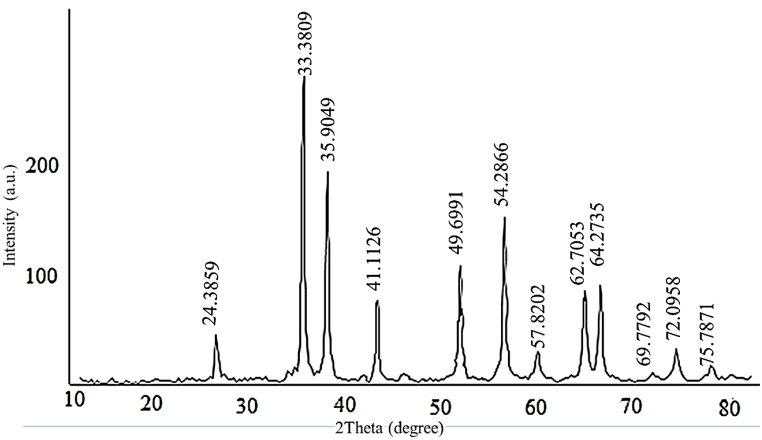
XRD plot of porous α-Fe_2_O_3_ NPs calcined at 350 °C.

The XRD spectra of mesoporous α- Fe_2_O_3_ NPs synthesized using rosemary extracts via hydrothermal technique is illustrated in Fig. 1. Consequently, the planes observed in the 24.2^0^ (012), (104), (110), (113), (024), (116), (018), (214), (300), (1010), and (220) are matched with the structure of α- Fe_2_O_3_ NPs (JCPDS data 01-072-0469) [41]. The intensity and presence of these peaks depend on the temperature and synthesis time. By increasing the temperature and duration of the hydrothermal process, the peaks became sharper, and the impurities disappeared, as reported in the literature [36]. The sharpest peak in (104) plane confirmed the hematite phase. Due to the area of the plane, hematite NPs had a rhombohedral (belonging to the space group *R*3*c*) structure [[42], [43], [44]]. Also, the average crystallite size (D) of porous hematite nanocarriers was calculated 31.60 nm by the Scherer's Debye equation ((D= (K × λ)/(β × COS θ)). In this equation, λ, θ and β factors are the wave length of the incident X-ray, diffraction angle, and the full-width at half maximum.

**Fig. 2:**
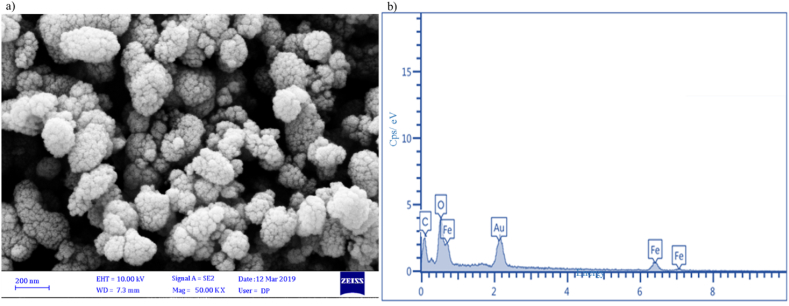
FESEM-EDS micrographs of porous α-Fe_2_O_3_ NPs calcined at 350 °C: **(a)** SEM images, and **(b)** EDS micrographs.

Fig. 2a displays the morphology and size of the synthesized porous α-Fe_2_O_3_ NPs calcined at 350 °C. In this micrograph NPs are evident with spherical, oval and pile flower-like shapes. The size of spherical and oval particles is randomly in the range of 26 nm–67 nm. On the other hand, the size of the pile flower-like is randomly in the range of 174 nm–369 nm. Washing and calcination of NPs has removed the elements and impurities in the final product. Also, calcination and phytochemicals in rosemary extract have caused the development of particles and the formation of pile flower-like [45]. Fig. 2b displays the existence of Fe, O and C elements with 50.5 wt%, 28.3 wt% and 21.2 wt%, respectively. The presence of C in the structure of prepared porous α-Fe_2_O_3_ corresponds to the polyphenols of rosemary extract. The plant precursor is a stabilising and reducing agent in the prepared of NPs [46,47]. The Au element in the EDS spectrum is related to the preparation of nanoparticles by gold coating process.

**Fig. 3:**
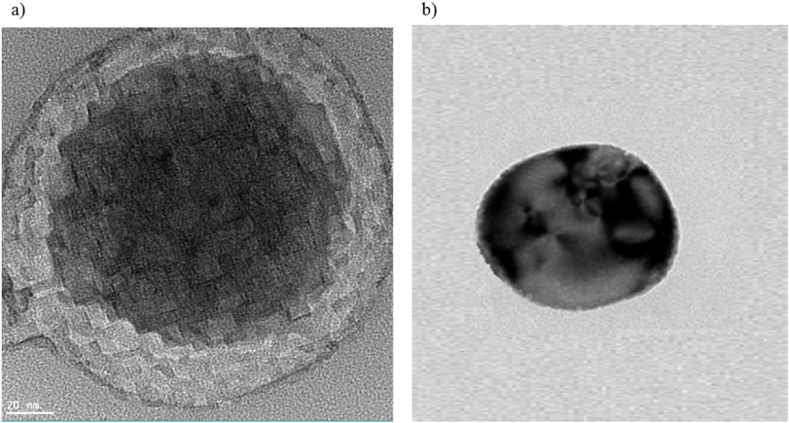
HR-TEM micrographs **(a)** hexagonal unit cell and **(b)** the surface pore entrances of the porous α-Fe_2_O_3_ NPs calcined at 350 °C.

HRTEM micrograph of porous α-Fe_2_O_3_ NPs calcined at 350 °C are shown in Fig. 3. The mesoporous fringes and lattice lines can be clearly identified inside the spherical hematite (α-Fe_2_O_3_) NPs, confirming the biogenic nanoparticles' mesoporous nature. As mentioned in the XRD analysis, these particles had a hexagonal crystal structure, and rhombic hexahedron nanocrystals are clearly visible in Fig. 3a. Additionally, Fig. 3a demonstrates the prepared HR-TEM image in a bright-field (BF) (scale: 20 nm) of single crystalline grains of porous hematite (α-Fe_2_O_3_) NPs. In this Figure, a hexagonal NP is shown with nearly almost spherical NPs. As shown in Fig. (3a) and (3b), a local reduction of the NPs thickness caused holes in the surface or inside of the NPs. As a result, the dark areas in Fig. (3b) illustrates agglomeration and alteration in wall thickness of the spherical hematite NPs [48,49].

**Fig. 4:**
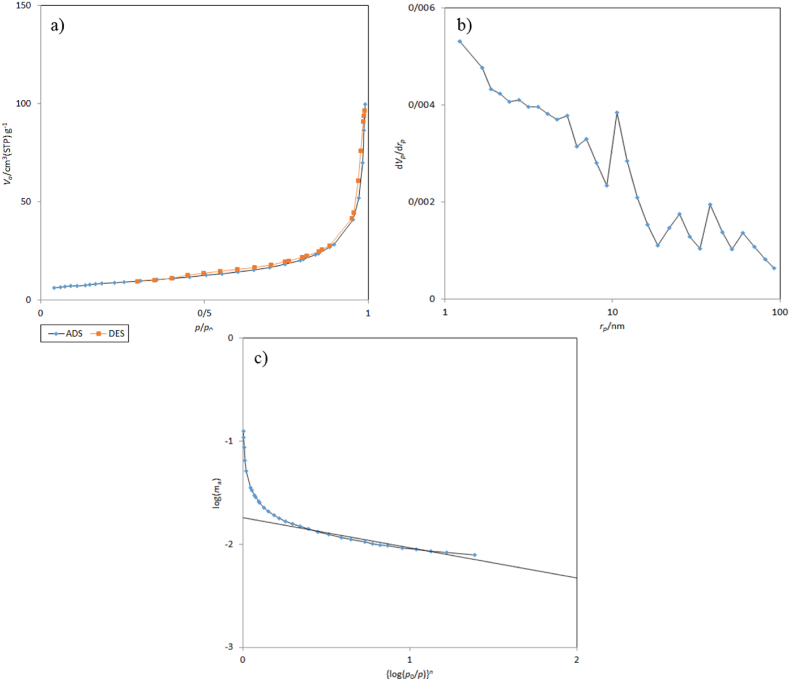
Nitrogen adsorption-desorption isotherms (**a**), BJH plot (**b**), and DA plot (**c**) porous α-Fe_2_O_3_ NPs calcined at 350 °C.

The Porosity characteristic of porous α-Fe_2_O_3_ NPs ocarriers calcined at 350 °C was corroborated by BET analysis. Nitrogen adsorption and desorption micrographs confirm the poor hysteresis-3 type on the basis of the IUPAC classification (Fig. (4a)). Based on this hysteresis at p/p0 = 0.4–0.45, the synthesized NPs has soft and plate-like cavities. Also, type-IV isotherm confirms the existence of micro-mesoporous. The specific surface area, total pore volume, and the mean pore diameter of porous α-Fe_2_O_3_ NPs were 30.8 m^2^/g^−1^, 0.14 cm^3^g^-1^, and 18.9 nm, respectively. Fig. (4b) and (4c) exhibit the Barrett-Joyner-Halenda (BJH) and Dubinin-Astakhov (DA) models for the pore size distributions of the porous NPs. DA plot shows that there are no micropores, while the BJH plot shows mesoporous compounds. Based on the BJH plot, the mesoporous pores range from 1.2 nm to 92.63 nm. Mesoporous pores are not uniform located and most pores are in the range of 2 nm–60 nm. According to the DA plot data, the pore size distribution at 2 nm was due to the uniform NPs aggregation together in the pile flower-like area (according to the SEM images). Thus, results obtained from these three plots confirmed that these biogenic NPs were mesoporous [36,50].

**Fig. 5:**
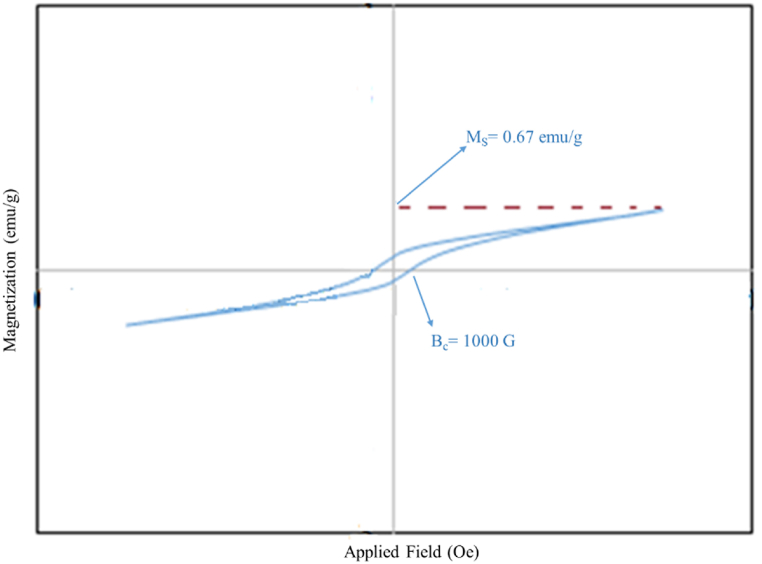
VSM graph of porous α-Fe_2_O_3_ NPs calcined at 350 °C.

The magnetic characterization of mesoporous hematite (α-Fe_2_O_3_) NPs calcined at 350 °C could be obtained through VSM analysis at room temperature (Fig. 5). The magnetic properties of hematite NPs depend on the synthesis approach, their calcination temperature, size, and shape [51,52]. According to the literature, bulk hematite NPs were weak ferromagnets; spherical hematite NPs with a size of <50 nm were superparamagnetic. Additionally, increasing the NPs calcination temperature enhanced the saturation magnetization (Ms) [53]. As shown in Fig. 5, low-grade calcined NPs have increased coercivity (Hc) and decreased saturation magnetization (Ms). According to the SEM results, the size of NPs was increased, and the magnetic property was decreased due to the accumulation of NPs in some areas; the weak ferromagnetic behavior could be detected in NPs with the size of about 100 nm [52]. Also, the presence of Rosemary extract compounds as coating agents around the metal ions reduced the surface moments and magnetization saturation (Ms) of the mesoporous hematite NPs [5]. Therefore, the weak magnetic properties of the nanocarrier are desirable for gene delivery applications on account of larger magnetic properties form aggregates after exposure to a magnetic field. The studies of Kashkouli et al. showed that the application of external magnetic field in gene transfer to HEK-293T cells increased the efficiency of gene transfer by 45%. This efficiency in gene transfer to these cells without applying an external magnetic field was 15%. Also, in this study, magnetic amino functionalized chitosan nanoparticles have weak magnetic properties due to chitosan coating [40].

**Fig. 6:**
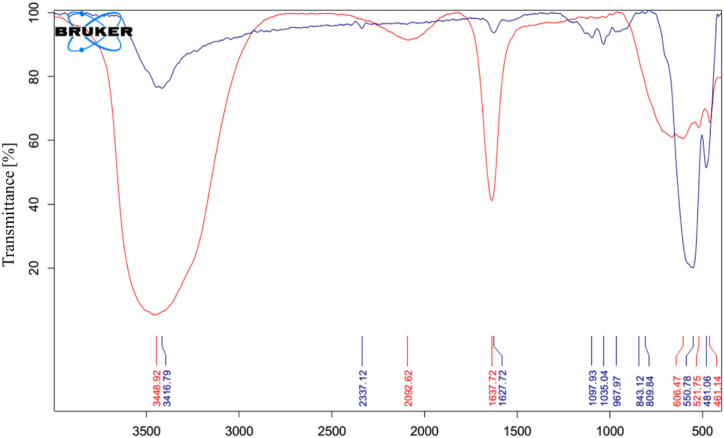
FTIR spectrum of porous α-Fe_2_O_3_ NPs calcined at 350 °C.

Fig. 6 shows the surface groups of porous NPs (**blue band**) and rosemary extract (**red band**). The absorption peaks in the region of 3416.79 cm^−1^ and 3448.92 cm^−1^ respectively matches to the stretching bond in NPs and the free functional group of -OH polyphenols in rosemary extract. The decrease peak in this area in the NPs confirmed the coating and reducing role of rosemary polyphenols. The broad peaks of 1637.72 cm^−1^ and 461-606 cm^−1^ in rosemary extract correspond to C=O stretching vibration and C-C- stretches in aromatic ring respectively [[54], [55], [56]]. The wide absorption spectrum in the region of 550.78 cm^−1^ and 481 cm^−1^ in the graph of synthesized NPs corresponds to Fe-O stretching bond and O-Fe-O bending bond, respectively. The minor absorption at 1627.72 cm^−1^ region of the nanoparticles matches to the C=O bending bond. The reduction of the peak of the extract in the same area confirmed its presence in the structure of NPs [57,58].

**Fig. 7:**
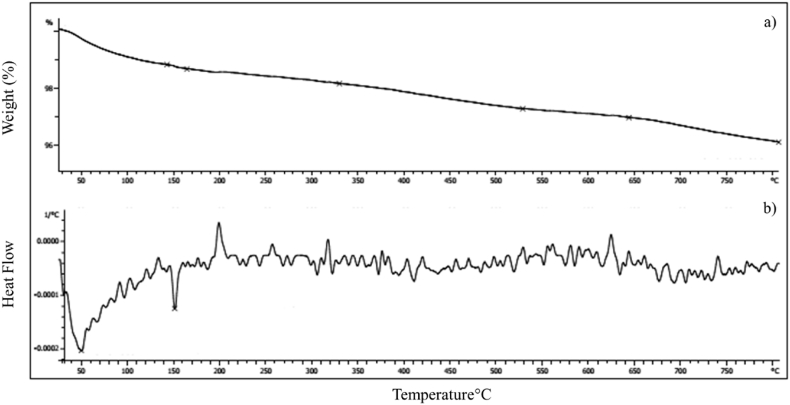
TGA-DTG curves of porous α-Fe_2_O_3_ NPs calcined at 350 °C.

TGA-DTG measurements of porous α-Fe_2_O_3_ NPs calcined at 350 °C are shown in Fig. 7. In this Figure, the weight loss is percentage as a function of temperature. Based on TGA micrograph, the total weight loss of porous α-Fe_2_O_3_ NPs is 3.91% (11.3106 mg) (Fig. 7a). The insignificant weight loss of the synthesized nanoparticles confirmed its thermal stability. The partial weight loss of 1.35% at 25 to 153 °C matches to the loss of NPs surface water. According to the DTG micrograph, the peak of endothermic in this range is at a temperature of 50 °C (Fig. 7b). Also, a partial endothermic peak is evident at 150 °C. The weight loss of 1.7% in the temperature range of 650-150 °C confirms the crystallization and phase transition of hematite NPs. In other words, weight loss in this temperature range confirms the transformation of Fe^3+^ ion and the formation of hematite phase [59,60]. A small weight loss of 0.05% in the temperature range of 650–800 °C is related to the removal of excess organic matter. The partial weight loss of the synthetic nanoparticle confirmed the stability and absence of impurities in the structure of the synthetic nanoparticle. Aggregation and agglomeration of NPs reduces the active sites for gene loading. Agglomeration of NPs is due to the presence of carbon in the polyphenols of the extract. Weight loss at a temperature higher than 650 °C causes the removal of the carbonaceous material of the extract and the creation of mesopores in the interior of the crystals.According to the BET results, these internal structures correspond to pores of 1.2 nm to <4 nm. These interior structures increase the active site and gene loading [61].

### 3.2 In vitro cytotoxicity and gene delivery

**Fig. 8:**
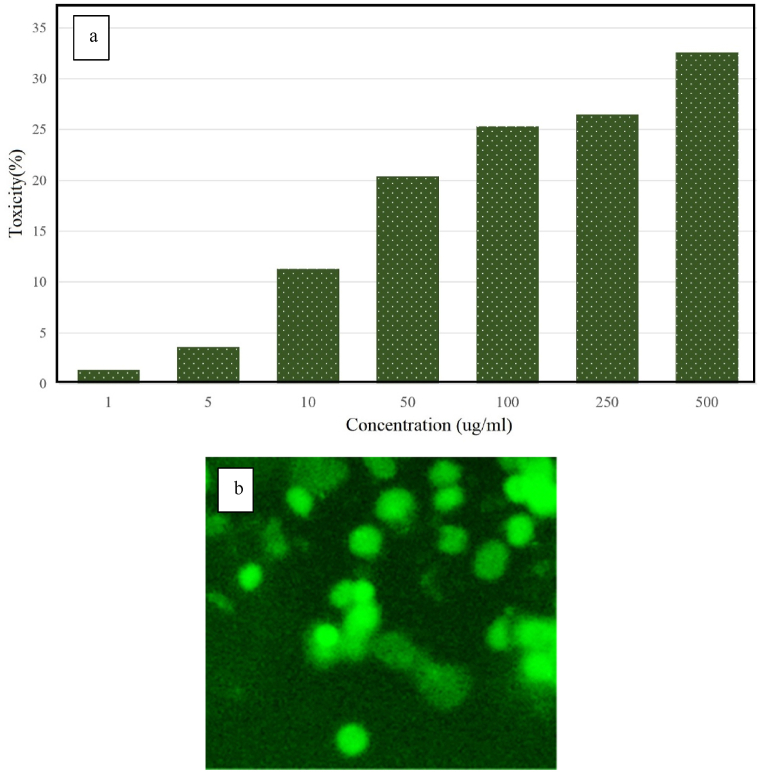
Cytotoxicity of different concentrations of porous α-Fe_2_O_3_ NPs against HEK-293T cell (**a**) and (**b**) gene delivery micrograph of porous α-Fe_2_O_3_ NPs calcined at 350 °C on HEK-293T cell line.

One of the most important features of carriers used in gene therapy is their safe nature and cellular biocompatibility. Therefore, biogenic nanocarriers are superior to other carriers, including viral carriers and chemical nanocarriers. Cytotoxicity of biogenic NPs was evaluated synthesized in different concentrations (1, 5, 10, 50, 100, 250, 500 μg/ml) for 24 h on cells HEK-293T with MTT method. 500 μg/ml, 250 μg/ml and 100 μg/ml of porous α-Fe_2_O_3_ NPs caused a 32.6%, 26.5% and 25.3% decrease in HEK-293T cells, respectively (Fig. 8a). The inhibition rate of these green NPs in the concentration of 1 μg/ml, 5 μg/ml and 10 μg/ml, 50 μg/ml is 1.4%, 3.6%, 11.3% and 20.4%, respectively. While in the reports of wilkinson et al., the toxicity of non-biogenic α-Fe_2_O_3_ NPs against HEK-293T cells at a concentration of 10 μg/ml is significant [62]. The results showed that the biogenic nanocarrier had little toxicity in concentrations of 1–500 μg mL^−1^. Therefore, the α-Fe_2_O_3_ biogenic nanocarrier as the best carrier reduces the body's immune response and provides a good gene delivery with low toxicity in the biological system. Evaluation of *in vitro* gene delivery of biogenic nanocarriers to HEK-293T cells was done using fluorescent microscope. Fig. (8b) shows cells transfected with nanocarriers by with the external magnetic field. The bright and prominent points in the image confirm the expression of the GFP gene and the production of green protein in Heck cells. Khoshnevis et al. showed that water-base double-layer functionalized Fe oxide NPs at a concentration of 0.001 μg mL had the least toxicity against EK293 cell lines. Also, after 24 h of HEK293 cell transfection, the highest protein expression was observed with magnetic induction [63]. Mesoporous iron NPs have no toxicity to Rat MSCs. The porosity of the NPs has resulted in a favorable loading. As a result, the gene delivery into the stem cells was successful [64]. Hidalgo et al. proved that iron (iii) carboxylate metal-organic nanocarriers successfully delivered siRNA into SW480 cells [65].

The *in vitro* studies of this research showed that biogenic NPs have negligible toxicity compared to their non-biogenic counterparts. Also, these biogenic particles have the ability to gene delivery to cells with the same efficiency as non-biogenic NPs.

Nowadays, the safety of the carriers, the optimal loading of the gene/drug and sustained release systems have led to the design of multifunctional biological systems. Non-viral carriers designed with plant extracts, organic materials, biopolymers, etc. have been considered due to the low risk of infection, low immunogenicity, flexibility of the structure, and ease of design. On the other hand, the low transmission efficiency of non-viral carriers is compensated by the porous nature and multimetallicof their structure. The covering role of extracts and organic substances in reducing metal ions has reduced the toxicity of metal ions. Porosity and specific shapes of nanocarriers (rods, bimetals, multimetallic, flowers, etc.) have caused many junctions and increased loading efficiency. Increasing biocompatibility and loading has increased the efficiency of transmission by biogenic systems. In addition, the presence of internal microcavities has caused the slow and continuous release of the gene/drug. Also, there are different methods for the synthesis of α-Fe_2_O_3_ nanoparticles, including microwave–hydrothermal [66], chemical vapor deposition [67], sol–gel process [68], microwave-assisted [69], electrodeposition [70], sonochemical [71] and co-precipitation methods [72]. These processes require numerous equipment and precursors that leave toxic byproducts. The green synthesis method does not require many steps and precursors and equipment. Also, the presence of herbal extract with therapeutic properties as a coating agent has reduced the toxicity of the nanocarrier. There are few studies of biogenic nanocarriers in gene therapy systems. NiCoFe_2_O_4_ trimetallic nanoparticles with cubic structure and porous nature were produced by green synthesis method. This magnetic nanoflower had no significant toxicity in high concentration (250 μg mL^−1^). Also, as a biogenic nanocarrier, it has been successful in transferring the GFP gene to HEK-293 T cells (N/P = 2.5) [5].

## 4 Conclusion

The aim of this research was to evaluate the potential of hematite α-Fe_2_O_3_ green NPs for the *in vitro* transfection of eukaryotic cells. Green hematite nanocarriers were synthesized using an eco-friendly hydrothermal method in one step with aqueous rosemary extract. The porosity of the NPs causes superficial and intracavitary loading of the plasmid. As a result, loading efficiency increases. Rosemary extract has reduced and covered hematite particles. Calcination of NPs has caused the removal of impurities and their stability. In addition, rosemary polyphenols and calcination temperature have caused the growth of iron particles and the formation of pile flower-like shapes. The accumulation of NPs and the existence of flower-like areas have caused the reduction of the magnetic properties of NPs. Also, this accumulation has caused the formation of capillary pores. The biogenic nature of NPs has resulted in minimal toxicity and high biocompatibility. The magneticity of nanocarriers causes its targeted delivery. Therefore, synthetic porous α-Fe_2_O_3_ green NPs are suitable non-viral carriers for gene therapy.

## Competing financial interests

The authors confirm that there are no competing interests.

## Ethics approval

Not applicable.

## Funding

This research received no external funding.

## Consent for publication

Not applicable.

## Data availability statement

The data are available from the corresponding author on reasonable request.

## CRediT authorship contribution statement

**Hajar Q. Alijani:** Formal analysis, Data curation, Conceptualization, Writing - orginal draft. **Shahram Pourseyedi:** Resources, Project administration, Methodology, Investigation, Funding acquisition, Formal analysis, Data curation, Conceptualization. **Masoud Torkzadeh-Mahani:** Validation, Software, Project administration, Investigation, Data curation, Conceptualization. **Mehrdad Khatami:** Writing – review & editing, Writing – original draft, Validation, Resources, Project administration, Methodology, Investigation, Funding acquisition, Data curation, Conceptualization.

## Declaration of competing interest

The authors declare that they have no known competing financial interests or personal relationships that could have appeared to influence the work reported in this paper.
